# Identification of a novel microdeletion causative of Nance‐Horan syndrome

**DOI:** 10.1002/mgg3.1879

**Published:** 2022-02-05

**Authors:** Mariana Lopez Martinolich, Hope Northrup, Pedro Mancias, Paul Hillman, Kavya Rao, Kate Mowrey

**Affiliations:** ^1^ Department of Pediatrics Division of Medical Genetics McGovern Medical School at the University of Texas Health Science Center at Houston (UTHealth Houston) and Children’s Memorial Hermann Hospital Houston Texas USA; ^2^ Department of Pediatrics Division of Child and Adolescent Neurology McGovern Medical School at the University of Texas Health Science Center at Houston (UTHealth Houston) and Children’s Memorial Hermann Hospital Houston Texas USA

**Keywords:** microdeletion, Nance‐Horan syndrome, *NHS*

## Abstract

**Background:**

Nance‐Horan syndrome (NHS) is a rare X‐linked genetic disorder characterized by ophthalmologic and dental anomalies as well as dysmorphic facies. The clinical phenotype in males includes congenital cataracts, vision loss, microcornea, nystagmus, microphthalmia, glaucoma, screwdriver blade‐shaped incisors, supernumerary maxillary incisors, diastema, delays, intellectual disability, and dysmorphic facies. With the evolution of array‐CGH technology, a total of five kindreds with NHS have been reported in the medical literature with microdeletions encompassing the *NHS* gene rather than sequencing variants.

**Methods:**

The patient is a 19‐year‐old male born to non‐consanguineous parents with a past medical history of bilateral congenital cataracts, nystagmus, poor vision, glaucoma, screwdriver blade‐shaped incisors, global developmental delay, intellectual disability, bilateral sensorineural hearing loss, axial hypotonia, and bilateral foot contractures.

**Results:**

A chromosomal microarray (CMA) was performed and revealed a 1.83‐Mb interstitial microdeletion at Xp22.2p22.13 (16,604,890–18,435,836) (GRCh37/hg19) that included *NHS*, *CTPS2, S100G, TXLNG, RBBP7, REPS2, SCML1, RAI2,* and *SCML2*.

**Conclusion:**

Here, we report the second largest microdeletion causative of NHS which also encompasses the remaining four kindreds in hopes of offering a unique perspective at the clinical variability within NHS, investigate genes of interest, and expand the phenotype.

## Graphical abstract

Nance‐Horan syndrome (NHS) is a rare X‐linked genetic disorder characterized by ophthalmologic and dental anomalies as well as dysmorphic facies. Here, we report the second largest microdeletion causative of NHS which also encompasses the remaining four kindreds in hopes of offering a unique perspective at the clinical variability within NHS, investigate genes of interest, and expand the phenotype.

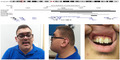

## 1 INTRODUCTION

Nance‐Horan syndrome (NHS) (OMIM 302350) is a rare X‐linked disorder that is characterized by ocular and dental anomalies as well as dysmorphic facies. Hemizygous male patients with this syndrome often present with a more pronounced phenotype than their heterozygous female counterparts (Coccia et al., 2009). The clinical phenotype in affected males includes congenital cataracts, microcornea, nystagmus, microphthalmia, glaucoma, screwdriver blade‐shaped incisors, diastema, developmental delays, and intellectual disability (Accogli et al., 2017). In contrast, heterozygous females experience a milder and more varied phenotype including posterior Y sutural cataracts, microcornea, and the characteristic dental abnormalities (Coccia et al., 2009). To date, an estimated 36 affected families have been identified within the literature.

The causative gene for Nance‐Horan syndrome is *NHS*. *NHS* was mapped to Xp21.1‐p22.3 by Stambolian et al. (1990) and determined to be the etiology of NHS by Burdon et al. (2003). Most pathogenic variants involve frameshift or nonsense mutations in the *NHS* gene that result in the truncation of the NHS protein (Accogli et al., 2017). Although the exact gene function remains unknown, the *NHS* gene is highly expressed in the midbrain, retina, lens, and teeth and its corresponding protein is implicated in the regulation of brain, teeth, and eye development. The gene spans ~650 Kb and includes 10 exons (Liao et al., 2011).

With the evolution of array‐CGH technology and its indication as a first‐tier genetic test for children with birth defects and developmental delays, five kindreds with NHS have been reported in the medical literature with microdeletions and/or complex genomic rearrangements encompassing the *NHS* gene (Accogli et al., 2017; Coccia et al., 2009; Liao et al., 2011; van Esch et al., 2007). These publications have suggested that the additional genes encompassed in these microdeletions could be influencing the phenotype and have been an area of recent interest.

In this report, we describe a 19‐year‐old man with a 1.83‐Mb interstitial microdeletion at Xp22.13, detected by chromosomal microarray (CMA), encompassing nine OMIM genes, including *CTPS2, S100G, TXLNG, RBBP7, REPS2, NHS, SCML1, RAI2*, and *SCML2*. Notably, this is the second largest microdeletion reported to date and encompasses the entirety of the microdeletions and complex genomic rearrangements observed in four previously reported kindreds (Accogli et al., 2017; Coccia et al., 2009; Liao et al., 2011).

## 2 CASE REPORT

The patient is a 19‐year‐old male born to non‐consanguineous parents at term via C‐section following a pregnancy complicated by gestational diabetes. Mother denies any prenatal exposures. While the parents did not receive a diagnosis of infertility, they report having had difficulties conceiving and never had additional children.

Patient birth weight was 3950 g (78th percentile); head circumference and length are unknown. At birth, the patient required the use of continuous positive airway pressure and remained in the neonatal intensive care unit until his third week of life. He was diagnosed with bilateral cataracts and nystagmus at birth and underwent cataract surgery at roughly 3 weeks old. He was diagnosed with glaucoma at 2 years and was subsequently prescribed eye drops for treatment and continues to follow with ophthalmology regularly. At the age of 7 years, the patient underwent an ophthalmologic exam under anesthesia that revealed the right eye was horizontally 9.5 mm and vertically 10.0 mm while the left eye was horizontally 10.0 mm and 10.0 mm vertically. Additionally, his left cornea was noted to be completely opacified, edematous, and had scleral thinning. The patient remains visually impaired and requires the use of corrective lenses. At age 3 years, an orchiopexy was performed to correct his undescended testicles. The parents report that the patient experienced frequent and recurrent ear infections. Per report, auditory brain stem response performed when he was ~4 years old identified bilateral sensorineural hearing loss. At age 6 years, bilateral ear tubes secondary to his recurrent otitis media.

A diagnosis of significant developmental delay and intellectual disability was made prior to school age and the patient received early childhood interventions in the form of weekly physical, occupational, and speech therapy. Unfortunately, psychometric evaluations characterizing his developmental delays, if performed, were not available to review by the authors. The patient remained in special education classes throughout his schooling and continues to receive therapies, although speech therapy was discontinued at age 17 years. The patient was noted to have bilateral foot contractures that have been present since childhood requiring orthotics in the past. He was evaluated by orthopedics at 18 years and was determined to have significant ankle osteoarthritis, forefoot deformity, and significant hindfoot valgus with subfibular impingement. His feet have become more fixed and flatter over the years. He can no longer walk for extended periods of time or place his feet together.

**FIGURE 1:**
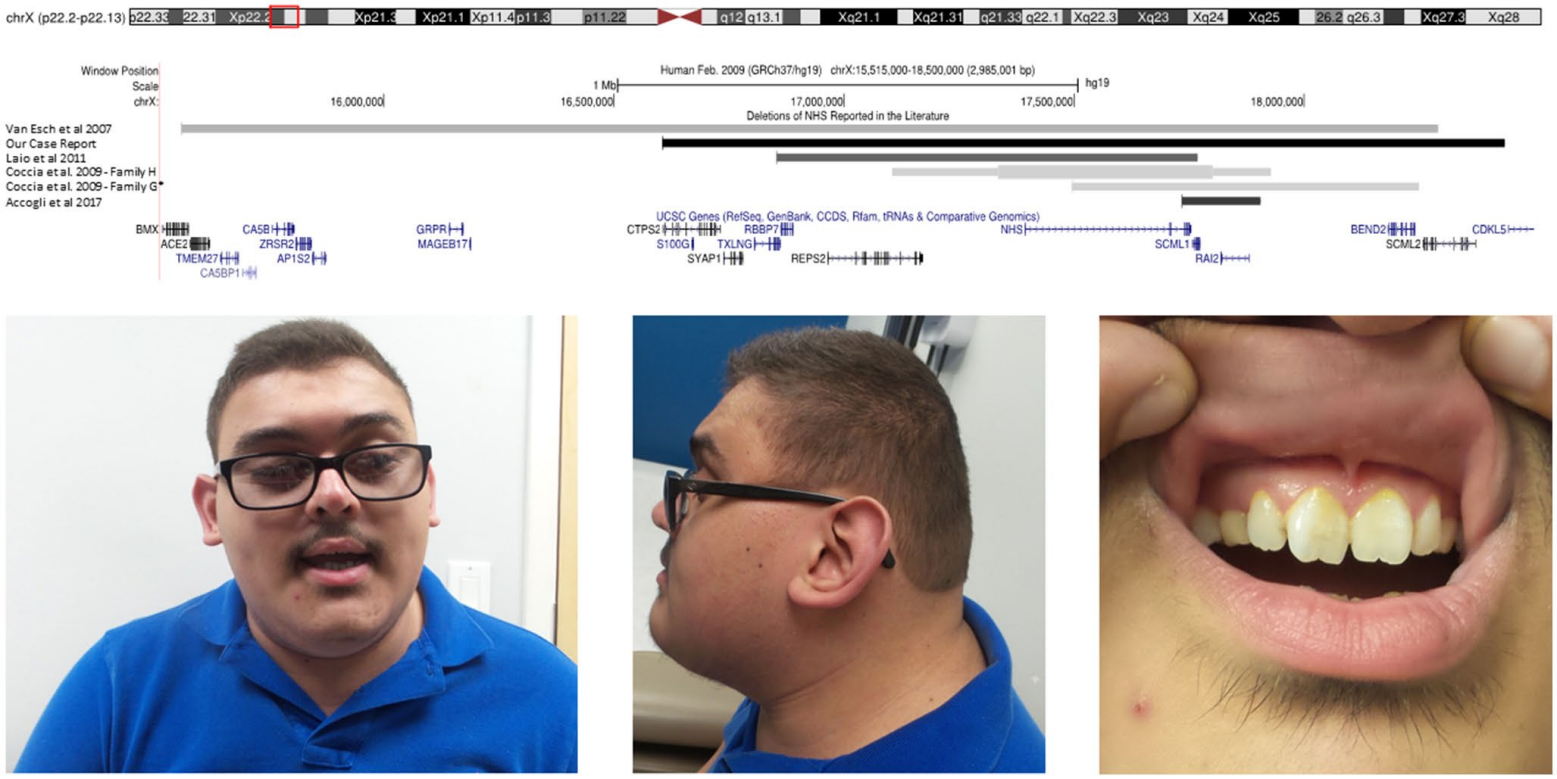
Top row: Comparison of microdeletions and genomic rearrangements in the five previously reported kindreds and our case (http://genome.ucsc.edu). Asterisk (*) indicates that the size of the deletion was estimated based on the information available in the original article. Thickened segment (Coccia et al., 2009) represents the reported triplication embedded within the indicated duplicated region. Bottom row: Front view, profile, and upper dental arch of our patient highlighting the classic dysmorphic features associated with Nance‐Horan syndrome

At age 18 years, the patient was evaluated by neurology secondary to his developmental delay and intellectual disability. During the neurology evaluation, he was also noted to have axial hypotonia and a wide based gait. A chromosomal microarray (CMA) was performed and revealed a 1.83‐Mb interstitial microdeletion at Xp22.2p22.13 (16,604,890–18,435,836) (GRCh37/hg19) that included the *NHS* gene, leading to a diagnosis of Nance‐Horan syndrome. The microdeletion also included the following genes: *CTPS2, S100G, TXLNG, RBBP7, REPS2, SCML1, RAI2*, and *SCML2*. He was subsequently referred to genetics who performed a detailed physical exam which noted obesity, brachycephaly, lateral deviation from midline of all toes, small hands, large anteverted ears bilaterally, microcornea, microphthalmia, chronic rotatory nystagmus, poor dentition with gray, and chipped teeth in addition to screwdriver blade‐shaped incisors and a high arched palate (Figure 1). A limited physical exam was performed on the mother and microcornea was observed, but maternal targeted testing for the Xp22.2 microdeletion was not performed due to financial constraints. A three‐generational pedigree was obtained and was noncontributory.

## 3 DISCUSSION

This case report describes the clinical features of the NHS patient with the second largest Xp22.13 microdeletion reported to date. The deletion in our patient encompasses the microdeletions and complex genomic rearrangements previously reported in the remaining four kindreds. This can be visualized in Figure 1 (Karolchik & Kent, 2002). This offers a unique opportunity to compare the phenotypes among the four kindreds (Accogli et al., 2017; Coccia et al., 2009; Liao et al., 2011). A full comparison of phenotype across the five kindreds with microdeletions can be seen in Table 1. The previously reported microdeletions have sparked interest in the roles of the other genes in that region and its potential for modifying the phenotype of NHS.

**TABLE 1:** Phenotype comparison among microdeletions causative of Nance‐Horan syndrome

Reported features of NHS	Our case	Mathys et al. (2007)	Accogli et al. (2017)	Liao et al. (2011) (II‐1)	Liao et al. (2011) (II‐2)	Coccia et al. (2009) (Family G)	Coccia et al. (2009) (Family H)
Eyes							
Congenital cataract	**✓**	**✓**	**✓**	**✓**	**✓**	**✓**	**✓**
Vision loss	**✓**						
Microcornea	**✓**	**✓**					
Nystagmus	**✓**		**✓**	**✓**			
Microphthalmia	**✓**	**✓**		**✓**		**✓**	
Glaucoma	**✓**						
Teeth							
Screwdriver blade‐shaped incisors	**✓**			**✓**	**✓**	**✓**	
Supernumerary maxillary incisors						**✓**	
Tapered premolar and molar cusps							
Diastema		**✓**	**✓**	**✓**	**✓**	**✓**	
Misalignment, discolored teeth		**✓**					
Neurologic							
Intellectual disability	**✓**			**✓**	**✓**		
Developmental delay	**✓**	**✓**	**✓**	**✓**	**✓**	**✓**	
Abnormal Behavior and/or autism	**✓**				**✓**		
Hypotonia	**✓**	**✓**	**✓**			**✓**	
Dysmorphic features							
Broad and/or Short fingers	**✓**				**✓**		
Prominent nose/nasal bridge	**✓**			**✓**	**✓**	**✓**	
Long, narrow face	**✓**			**✓**	**✓**	**✓**	
Large anteverted pinnae	**✓**		**✓**	**✓**	**✓**	**✓**	
Congenital heart defects		**✓**	X	X	X		**✓**
Skeletal							
Bilateral talipes planus				**✓**			
Bilateral hallux valgus				**✓**	**✓**		
Scoliosis					**✓**		
Pes planus	**✓**				**✓**		
Bilateral foot contractures	**✓**						
Forefoot deformity	**✓**						
Hindfoot valgus	**✓**						
Other							
Undescended testicles	**✓**			**✓**			
Sensorineural hearing loss	**✓**						

Abbreviations: **✓**, Feature present in patient; X, indicated as absent.

Coccia et al. (2009) reported on seven different families affected by NHS, but Family H was noted to have an atypical presentation for NHS. Family H was found to have a complex re‐arrangement consisting of a triplication of a region encompassing *NHS*, *SCML1*, and *RAI2* genes embedded within a duplicated region. In Family H, all six affected males had cataracts and no other clinical features of NHS. Four out of six affected males also had congenital heart defects, including ductus arteriosus, teratology of Fallot, ventricular septal defect, and stenosis of a major cardiac vessel. A cardiac defect was noted in Mathys et al. (2007) but have not been present in cases of NHS secondary to sequencing variants suggesting that flanking genes could be modulating the risk for cardiac defects (Mathys et al., 2007; van Esch et al., 2007). Although the patient in Mathys et al. (2007) had a larger microdeletion encompassing both *NHS* and *CDKL5*, which is known to cause CDKL5 deficiency disorder (CDD) (OMIM 300672), his secondary diagnosis does not explain the presence of the tetralogy of Fallot. Due to overlapping nature of the microdeletions reported thus far, Accogli et al. (2017) postulated that *RAI2* may play a role in cardiogenesis although the function of that protein is not well‐established at this time. Interestingly, our patient as well as the patients reported in Accogli et al. (2017), Family G in Coccia et al. (2009), and Liao et al. (2011) all have microdeletions that include *RAI2* and did not report a history of a cardiac defect. Notably, our patient did not have any symptoms that led to a cardiac evaluation.

In addition to the typical clinical features associated with NHS, Liao et al. (2011) reported skeletal features in their sibling pair, a clinical phenotype not appreciated in individuals with NHS with sequencing variants. In Liao et al. (2011), they identified a 0.92‐Mb microdeletion at Xp22.13 in two Taiwanese brothers encompassing *REPS2*, *NHS*, *SCML1*, and *RAI2* genes. The skeletal features noted in the elder brother was bilateral talipes planus and hallux valgus while the younger brother also had hallux valgus as well as scoliosis and bilateral pes planus. The skeletal findings in this sibling set are of interest as our patient demonstrated skeletal abnormalities, specifically bilateral pes planus, bilateral foot contractures, forefoot deformity, and hindfoot valgus. Skeletal differences in NHS have not been present in other previous reports, suggesting this may be an underreported feature or an expansion of the phenotype. Interestingly, *SCML1* and *RAI2* has been noted to be expressed in adult tissues in skeletal and heart, which could potentially account for the skeletal manifestations observed in our patient as well as Liao et al. (2011) (van de Vosse et al., 1998; Walpole et al., 1999).

Another unique feature identified in our patient is the presence of bilateral sensorineural hearing loss. Although our patient did have recurrent ear infections as a child, this would not lead to sensorineural hearing loss, but rather conductive hearing loss. No other patients with NHS have been reported to have sensorineural hearing loss. At this time, none of the genes included in our patient's microdeletion have been associated with ear development, notably inner ear development, or the associated nerve pathways. Therefore, this clinical finding may represent an expansion of phenotype and warrants continued research regarding the role of *NHS* and other flanking genes regarding ear development.

Our case report adds to the growing literature regarding the clinical presentation of NHS secondary to a microdeletion encompassing the *NHS* gene, highlights the inter‐familial variability previously described, and introduces clinical features that may represent an expansion of phenotype. This case report also emphasizes how the evolution of genetic technology and continued research into gene function holds the potential for clinicians to have a better understanding of the full spectrum of clinical presentations associated with genetic disorders, in hopes to enhance and improve the medical care for these patients and their families.

## CONFLICT OF INTEREST

The authors declare no financial or otherwise relevant conflict of interest related to this manuscript.

## Data Availability

The data that support the findings of this study are available on request from the corresponding author. The data are not publicly available due to privacy or ethical restrictions

## References

[mgg31879-bib-0001] Accogli, A. , Traverso, M. , Madia, F. , Bellini, T. , Vari, M. S. , Pinto, F. , & Capra, V. (2017). A novel Xp22.13 microdeletion in Nance‐Horan syndrome. Birth Defects Research, 109(11), 866–868. 10.1002/bdr2.1032 28464487

[mgg31879-bib-0002] Burdon, K. P. , McKay, J. D. , Sale, M. M. , Russell‐Eggitt, I. M. , Mackey, D. A. , Gabriela Wirth, M. , Elder, J. E. , Nicoll, A. , Clarke, M. P. , FitzGerald, L. M. , Stankovich, J. M. , Shaw, M. A. , Sharma, S. , Gajovic, S. , Gruss, P. , Ross, S. , Thomas, P. , Voss, A. K. , & Thomas, T. (2003). Mutations in a novel gene, NHS, cause the pleiotropic effects of Nance‐Horan syndrome, including severe congenital cataract, dental anomalies, and mental retardation. The American Journal of Human Genetics, 73, 1120–1130. 14564667 10.1086/379381 PMC1180491

[mgg31879-bib-0003] Coccia, M. , Brooks, S. P. , Webb, T. R. , Christodoulou, K. , Wozniak, I. O. , Murday, V. , Balicki, M. , Yee, H. A. , Wangensteen, T. , Riise, R. , Saggar, A. K. , Park, S. M. , Kanuga, N. , Francis, P. J. , Maher, E. R. , Moore, A. T. , Russell‐Eggitt, I. M. , & Hardcastle, A. J. (2009). X‐linked cataract and Nance‐Horan syndrome are allelic disorders. Human Molecular Genetics, 18(14), 2643–2655. 10.1093/hmg/ddp206 19414485 PMC2701339

[mgg31879-bib-0004] Karolchik, D. , & Kent, W. J. (2002). The UCSC genome browser. Current Protocols in Bioinformatics, 31(1), 51–54. 10.1002/0471250953.bi0104s40 23255150

[mgg31879-bib-0005] Liao, H. M. , Niu, D. M. , Chen, Y. J. , Fang, J. S. , Chen, S. J. , & Chen, C. H. (2011). Identification of a microdeletion at Xp22.13 in a Taiwanese family presenting with Nance‐Horan syndrome. Journal of Human Genetics, 56(1), 8–11. 10.1038/jhg.2010.121 20882036

[mgg31879-bib-0006] Mathys, R. , Deconinck, H. , Keymolen, K. , Jasen, A. , & van Esch, H. (2007). Severe visual impairment and retinal changes in a boy with a deletion of the gene for Nance‐Horan syndrome. Bulletin de la Societe belge d'ophtalmologie, 305, 49–56. 18018428

[mgg31879-bib-0007] Stambolian, D. , Lewis, R. A. , Buetow, K. , Bond, A. , & Nussbaumt, R. (1990). Nance‐Horan syndrome: Localization within the region Xp2I.I‐Xp22.3 by linkage analysis. American journal of human genetics, 47(1), 13. 1971992 PMC1683770

[mgg31879-bib-0008] van de Vosse, E. , Walpole, S. M. , Nicolaou, A. , van der Bent, P. , Cahn, A. , Vaudin, M. , Ross, M. T. , Durham, J. , Pavitt, R. , Wilkinson, J. , Grafham, D. , Bergen, A. A. B. , van Ommen, G.‐J. B. , Yates, J. R. W. , den Dunnen, J. T. , & Trump, D. (1998). Characterization of SCML1, a new gene in Xp22, with homology to developmental Polycomb genes. GENOMICS, 49, 96–102. 9570953 10.1006/geno.1998.5224

[mgg31879-bib-0009] van Esch, H. , Jansen, A. , Bauters, M. , Froyen, G. , & Fryns, J. P. (2007). Encephalopathy and bilateral cataract in a boy with an interstitial deletion of Xp22 comprising the CDKL5 and NHS genes. American Journal of Medical Genetics, Part A, 143(4), 364–369. 10.1002/ajmg.a.31572 17256798

[mgg31879-bib-0010] Walpole, S. M. , Hiriyana, K. T. , Nicolaou, A. , Bingham, E. L. , Durham, J. , Vaudin, M. , Ross, M. T. , Yates, J. R. W. , Sieving, P. A. , & Trump, D. (1999). Identification and characterization of the human homologue (RAI2) of a mouse retinoic acid‐induced gene in Xp22. http://www.sanger.ac.uk/HGP/ 10.1006/geno.1998.5667 10049581

